# A novel angiomatoid epithelioid sarcoma cell line, Asra-EPS, forming tumors with large cysts containing hemorrhagic fluid *in vivo*

**DOI:** 10.1186/1756-0500-6-305

**Published:** 2013-08-01

**Authors:** Yoshinori Imura, Norifumi Naka, Hidetatsu Outani, Hirohiko Yasui, Satoshi Takenaka, Ken-ichiro Hamada, Ritsuro Ozaki, Mitsunori Kaya, Ken-ichi Yoshida, Eiichi Morii, Akira Myoui, Hideki Yoshikawa

**Affiliations:** 1 Department of Orthopaedic Surgery, Osaka University Graduate School of Medicine, 2-2 Yamadaoka, Suita, Osaka 565-0871, Japan; 2 Department of Biology, Osaka Medical Center of Cancer and Cardiovascular Diseases, 1-3-3 Nakamichi, Higashinari-ku, Osaka 537-8511, Japan; 3 Department of Orthopaedic Surgery, Chitose City Hospital, 2-1-1 Hokko, Chitose, Hokkaido 066-8550, Japan; 4 Department of Pathology, Osaka University Graduate School of Medicine, 2-2 Yamadaoka, Suita, Osaka 565-0871, Japan

**Keywords:** Epithelioid sarcoma, Asra-EPS, VEGF, CA 125, INI-1, Cystogenesis

## Abstract

**Background:**

Whereas we can use several human epithelioid sarcoma (ES) cell lines for basic and preclinical research, an angiomatoid ES cell line has not been reported to date. We have treated a case of an angiomatoid ES developing in the right upper extremity of a 67-year-old man.

**Methods:**

An angiomatoid ES cell line, Asra-EPS was newly established and characterized for its morphology, growth rate and chromosomal analysis. Tumorigenicity of Asra-EPS cells was also analyzed in athymic nude mice.

**Results:**

Asra-EPS cells were round, polygonal or spindle-shaped with an abundant cytoplasm and have been maintained continuously *in vitro* for over 150 passages during more than 15 months. These cells secreted cancer antigen 125 (CA 125), interleukin-6 (IL-6) and vascular endothelial growth factor (VEGF) into the culture medium. Asra-EPS cells were tumorigenic when implanted in nude mice with tumors reaching a volume of 1000 mm^3^ at around 50 days. Histological features of tumors formed in mice were essentially the same as those of the original tumor, exhibiting a multinodular proliferation of eosinophilic epithelioid and spindle-shaped cells with prominent areas of hemorrhage and blood-filled cystic spaces strikingly corresponding to the potential of hemorrhagic cyst formation in the original tumor. They showed immunopositive staining for cytokeratins (AE1/AE3 and CAM5.2), epithelial membrane antigen (EMA), vimentin, CD31, CD34 and CA 125, but negative for integrase interactor 1 (INI-1) and factor VIII-related antigen.

**Conclusions:**

The established cell line represents a biologically relevant new tool to investigate the molecular pathology of human angiomatoid ES and to evaluate the efficacy of novel therapeutics both *in vitro* and *in vivo*.

## Background

Epithelioid sarcoma (ES) was first described in 1970 by Enzinger as a distinct soft tissue tumor with mixed epithelial and mesenchymal phenotype, but the origin and true nature of ES remain controversial [1]. In general, ES is a relatively rare sarcoma and accounts for less than 1% of all soft tissue sarcoma [2]. The clinical course of ES is usually characterized by local recurrence and the potential for metastatic spread mostly to lymph nodes and lung, but effective chemotherapy has not been established [3,4]. Two main clinicopathologic types of ES are recognized. The classic type manifests clinically as a painless, slow-growing nodule arising within the dermis or subcutis or, less commonly, in deep fascial or tenosynovial tissue [4]. Usually, it occurs in young adults at the flexor surface of the fingers, hand, wrist, and forearm, followed by knee and lower leg. On the other hand, the less common type, also known as the proximal type or large cell/rhabdoid ES, occurs predominantly in middle-aged or older adults in axial proximal locations such as pelvis, mediastinum and trunk, as deep infiltrative masses [5]. Additionally, other lesser common variants have been described, including the angiomatoid variant and the fibroma-like variant [6,7]. Until now, some case reports of angiomatoid ES, which distinctively features cyst formation and hemorrhage within tumor nodules, have been described [8,9]. To date, a small number of ES cell lines have successfully been established [10-21], but there is no cell line from angiomatoid ES reported (Table 1). We herein present a case of angiomatoid ES arising in a 67-year-old man and establish an angiomatoid ES cell line from this patient. In this study, we describe the characteristics of a newly established angiomatoid ES cell line, Asra-EPS, both *in vitro* and *in vivo*.

**Table 1:** Clinical and cytogenetic findings in epithelioid sarcoma cell lines which have been established until now

**Cell line (year)**	**Age/sex**^**1**^	**Primary location**	**Doubling time**^**2**^	**Tumorigenicity**^**3**^	**CA125**^**4**^	**INI-1**^**5**^
RM-HS1 (1987)	37/M	Left foot	N.A	N.A	N.A	N.A
HX 165c (1988)	28/M	Penis	38 h	N	N.A	N.A
GRU-1 (1990)	32/M	Left buttock	29 h	N	N.A	N.A
SARCCR2 (1993)	33/F	Knee	28 h	N	N.A	N.A
ES020488 (1993)	26/M	Left forearm	60 h	N	N.A	N.A
VAESBJ (1995)	41/M	Epidural space	32 h	S	-	-
ES-OMC-MN (1997)	44/F	Right leg	68.5 h	-	N.A	N.A
YCUS-5 (1999)	3/F	Neck	N.A	N.A	N.A	N.A
SFT-8606 (2000)	75/M	Left elbow	N.A	S	+	N.A
FU-EPS-1 (2005)	21/M	Right upper arm	45 h	S	+	N.A
NEPS (2010)	32/M	Forearm	N.A	S	-	N.A
Epi-544 (2011)	N.A/N.A	Foot	48 h	S	+	-
Asra-EPS (2013)	67/M	Right elbow	38 h	N	+	-

^1^*F* female, *M* male. ^1 2 3 4 5^*N.A* data not available. ^3^*N* nude mice, *S* SCID mice.

## Methods

### Case report

**Figure 1:**
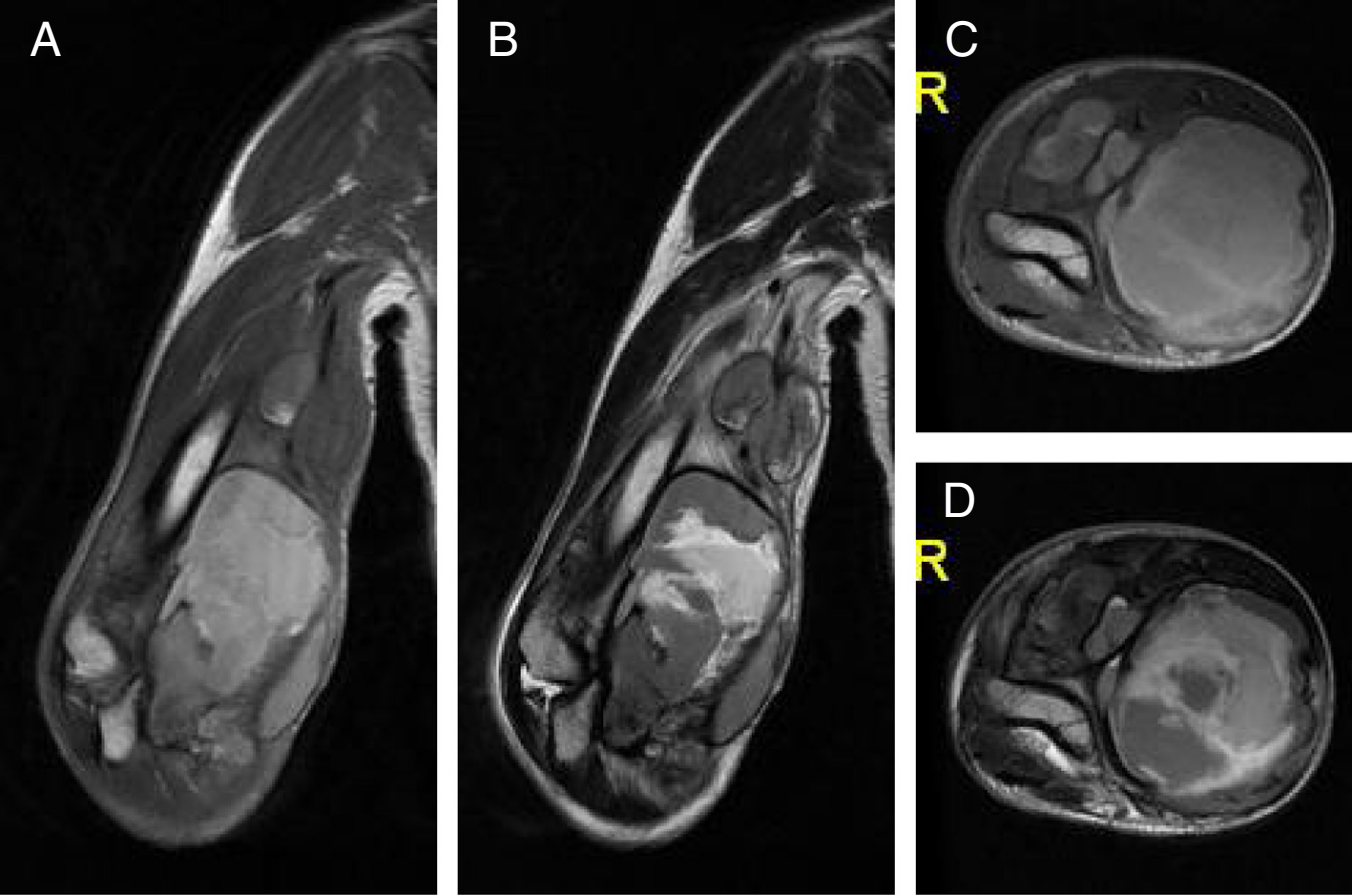
**MRI of the huge tumor in the right upper extremity of a 67-year-old man. (A)** T1-weighted coronal image. **(B)** T2-weighted coronal image. **(C)** T1-weighted axial image. **(D)** T2-weighted axial image.

**Figure 2:**
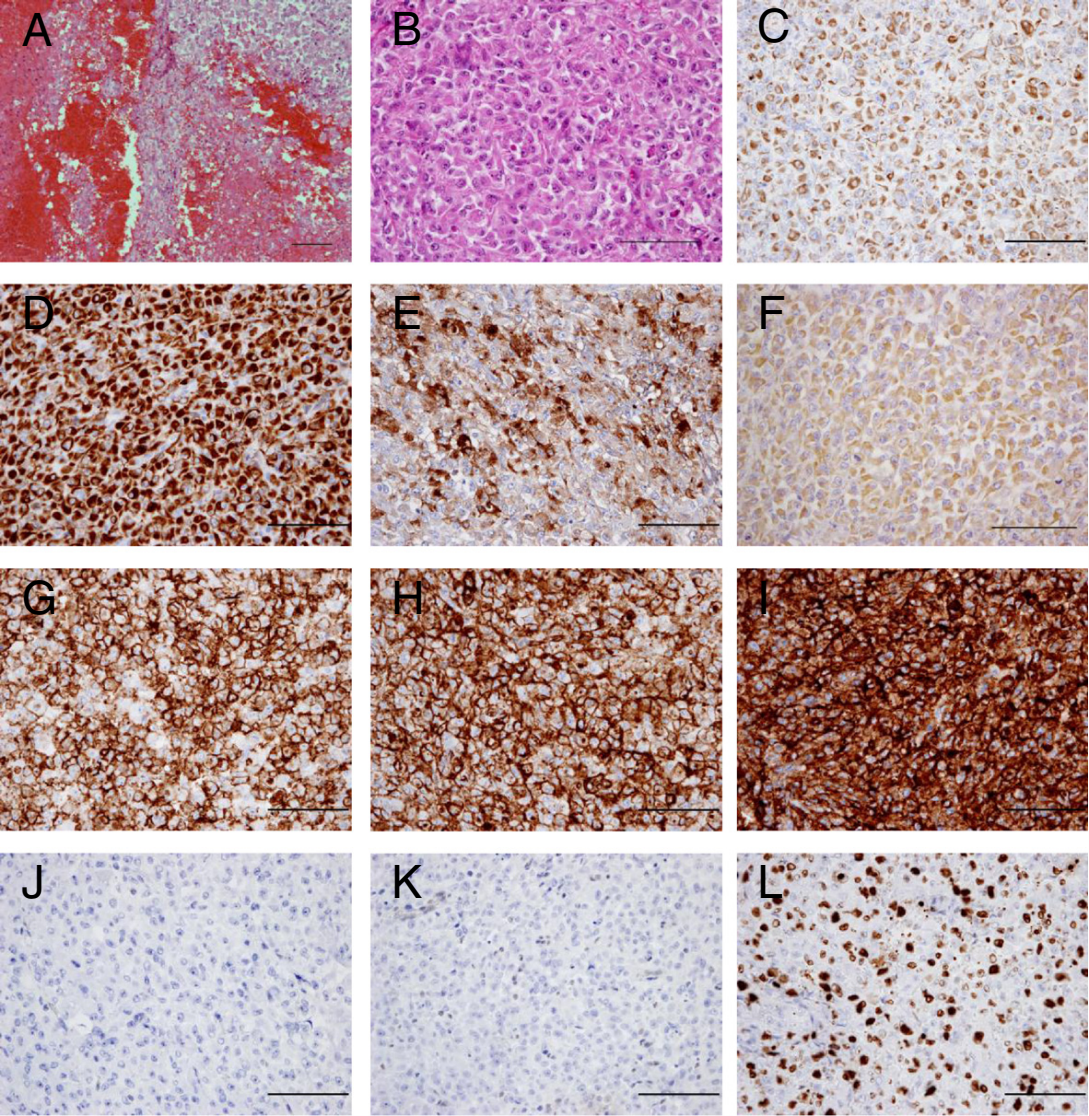
**Microscopic features of the patient’s original tumor. (A, B)** Histological appearance of the original tumor. H&E staining showed a multinodular proliferation of eosinophilic epithelioid and spindle-shaped cells with prominent areas of hemorrhage. **(C-L)** Immunohistochemical reactivity in the original tumor. Tumor cells were diffusely positive for AE1/AE3 **(C)**, CAM5.2 **(D)**, EMA **(E)**, vimentin **(F)**, CD31 **(G)**, CD34 **(H)** and CA 125 **(I)**, but negative for INI-1 **(J)** and factor VIII-related antigen **(K)**. Positive rate of Ki-67 was approximately 50% **(L)**. Scale bars: 100μm.

The patient was a 67-year-old Japanese man. Six months before presentation to our institute, he noted a painful soft tissue mass in his right elbow and was referred to the primary hospital. He had no history of trauma or other illness including hemorrhagic diathesis. The tumor was diagnosed as a spontaneous hematoma since magnetic resonance imaging (MRI) revealed fluid-fluid level collection. It had gradually increased in size over a 5-month period. Pain was activity related initially and became more constant later in the course. At the first presentation to our hospital, he had a huge tumor measuring 10 × 7 × 13 cm in his right upper extremity, with intolerable pain, numbness, and nearly complete palsy at the area of median and radial nerve in his hand. Blood analyses revealed the data to be within normal limits except for a marked elevation of C-reactive protein (CRP) and cancer antigen 125 (CA 125). The serum CRP and CA 125 levels were 7.52 mg/dl (normal: ≤ 0.2 mg/dl) and 769 U/ml (normal: ≤ 35 U/ml), respectively. Re-examined MRI showed large multinodular cystic tumors with satellite lesions in his right arm, infiltrating major neurovascular structures (Figure 1A, B, C, D). Computed tomography (CT) of the chest revealed no metastatic lesions in the lungs. Percutaneous needle biopsy was performed and microscopic examination of the specimen exhibited a nodular growth pattern with central necrosis, composed of compact sheets of epithelioid and spindle-shaped cells. In parts, prominent areas of hemorrhage and foci with blood-filled cystic spaces were observed. A tentative histological diagnosis of epithelioid angiosarcoma or angiomatoid ES was made. A right above-elbow amputation was performed because of high-grade malignancy and tumor infiltration into major neurovascular structures. Macroscopically, large cyst filled with coagulated blood occupied most part of the amputated arm. Microscopic examination of the specimen revealed the same as percutaneous needle biopsy (Figure 2A, B). Immunohistochemistry showed positivity of tumor cells for cytokeratins (AE1/AE3 and CAM5.2) (Figure 2C, D), epithelial membrane antigen (EMA) (Figure 2E), vimentin (Figure 2F), CD31 (Figure 2G), CD34 (Figure 2H) and CA 125 (Figure 2I) whereas integrase interactor 1 (INI-1) and factor VIII-related antigen were negative (Figure 2J, K), confirming the histological diagnosis as angiomatoid ES based mainly on the CA 125 and INI-1 immunoreactivity. Positive rate of Ki-67 was approximately 50% (Figure 2L). Radiotherapy composed of 60Gy/30fr was done between the right shoulder including axilla and the stump of arm postoperatively in 5 weeks. Adjuvant chemotherapy was not administered due to age based limitations. At 21 days after the operation, the serum CRP and CA 125 levels decreased to 0.03 mg/dl and 53 U/ml, respectively. Four months later, concomitant with re-elevation of the serum CRP and CA 125 levels, left axillary lymph node metastasis and pulmonary lymphangitis sarcomatosa were found. Six months after the primary surgery, the patient died of respiratory insufficiency.

### Establishment of a new cell line from an angiomatoid ES

Tumor cells were isolated from surgically resected tissues with the patient’s informed consent and under the guidelines of the Institutional Review Board for Clinical Research at Osaka University Hospital. The tumor tissues were minced and incubated with 1 mg/ml of collagenase (Sigma-Aldrich, St. Louis, MO, USA) for 1 h at 37°C. Cell suspensions were passed through a 40-μm nylon mesh (Becton Dickinson Falcon, Franklin Lakes, NJ, USA) and the tumor cells were cultured in Dulbecco’s modified Eagle’s medium (DMEM; GIBCO, Grand Island, NY, USA) with 10% fetal bovine serum (FBS; Sigma). The adherent cells were maintained for over 15 months in culture, and passed more than 150 times. Attached cells exhibited spindle-shaped, epithelioid and polygonal morphology. This cell line was named Asra-EPS. Asra-EPS cells were cultured at 37°C with 95% air, 5% CO_2_, and 100% humidity.

### Cell growth assay

To determine the doubling time, 1 × 10^5^ Asra-EPS cells were seeded into each well of 6-well plates (BD Falcon) with 2 ml DMEM, supplemented with 10% FBS. Three wells were trypsinized with 0.25% Trypsin plus EDTA (Invitrogen, Carlsbad, CA, USA) every 24 h and cell number for each well was counted with a hemocytometer for 8 days.

### Soft agar colony formation assay

To determine the anchorage-independent cell growth ability, soft agar colony formation assay was performed. Asra-EPS cells (5 × 10^3^) were added to 0.5% SeaPlaque Agarose (Lonza, Basel, Switzerland) in 1 ml DMEM with 10% FBS over 0.6% agar in 2 ml DMEM with 10% FBS in 35 mm plates (BD Falcon). For 3 weeks, plates were looked at every day to make sure they have not dried out and add growth medium to the plates when necessary.

### CA 125, interleukin-6 (IL-6) and vascular endothelial growth factor (VEGF) measurement *in vitro*

Resuspension of 1 × 10^5^ cells were planted in 2 ml DMEM supplemented with 10% FBS on 6-well plates. At 7th day, culture medium was collected and centrifuged at 1200g for 5 min to remove floating cells, and then the supernatant was stored at −80°C until assay. The concentration of CA 125, IL-6 and VEGF was measured by the chemiluminescent enzyme immunoassay (CLEIA) technique (SRL Inc., Tokyo, Japan).

### Nude mice xenografting

Five-week-old athymic nude mice (BALB/c nu/nu; SLC, Shizuoka, Japan) were housed at the Institute of Experimental Animal Sciences Osaka University Medical School, in accordance with guideline approved by the Institutional Animal Care and Use Committee of Osaka University Graduate School of Medicine. To determine the tumorigenicity of Asra-EPS cell line *in vivo*, 1 × 10^7^ cells were injected subcutaneously into the left side of back. Mice were inspected twice a week and were sacrificed when the total tumor burden reached 2 cm^3^. Tumor size was measured with a caliper and tumor volume calculated by the formula (a × b^2^)/2, with a being the longest diameter and b the shortest diameter of the tumor. Tumors were then resected for immunohistochemical studies.

### Immunohistochemistry

Immunohistochemical studies were performed to confirm whether the phenotype of the cultured cells matched with that of the original tumor cells. Specimens of the original tumor and of the tumors formed in nude mice were fixed in 10% neutral, buffered formalin, embedded in paraffin, sectioned at 4 μm thickness, and stained with hematoxylin and eosin (H&E). Paraffin-embedded sections were deparaffinized and dehydrated. Antigens were retrieved at 95°C for 15 min in 10 mM citrate buffer. After blocking of endogenous peroxidase activity for 10 min with methanol containing 3% H_2_O_2_, the sections were reacted for 1 h with phosphate-buffered saline (PBS) containing 2% bovine serum albumin (BSA) at room temperature to prevent nonspecific binding. They were then incubated overnight with primary antibodies at 4°C. Primary antibodies against AE1/AE3 (1:100, Dako, Glostrup, Denmark), CAM5.2 (BD Biosciences, San Jose, CA, USA), EMA (1:100, Dako), vimentin (1:100, Abcam, Cambridge, UK), CD31 (1:50, Dako), CD34 (1:100, Leica Microsystems, Wetzlar, Germany), CA 125 (1:50, Dako), INI-1 (1:50, BD Biosciences), factor VIII-related antigen (1:50, Dako) and Ki-67(1:75, Dako) were used. On the next day, the sections were incubated for 1 h with horseradish peroxidase (HRP)-conjugated secondary antibodies (1:500, BD Biosciences) and stained with 3,3′-diaminobenzidine tetrahydrochloride (DAB; Dako). The sections were finally counterstained with hematoxylin. As a negative control, staining was carried out in the absence of primary antibody.

### Western blotting

Equal amounts of protein extracted from cultured cells were separated on 4-12% Bis-Tris gels (Invitrogen) and transferred to PVDF membranes. The membranes were rinsed in Tris-buffered saline containing 0.1% Tween (TBS-T) and incubated in blocking buffer (5% skim milk in TBS-T) at room temperature. The blocked membranes were incubated with anti-INI-1 antibody (1:1000) and anti-beta-actin antibody (1:1000, Santa Cruz Biotechnology, Inc., Santa Cruz, CA, USA) at 4°C overnight and washed with TBS-T 3 times for 5 min each time, followed by incubation with HRP-conjugated second antibodies (1:2000) at room temperature for 1 h. After washing in TBS-T, the immunoreactive bands were visualized using enhanced chemiluminescence (ECL) (Perkin Elmer Life Sciences, Waltham, MA, USA).

### Chromosome analysis

Metaphase chromosome spreads from Asra-EPS cells were prepared according to standard procedures. Asra-EPS cells were treated with 20 μg/ml of colcemide overnight and harvested. After treatment of 0.075 M KCl for 20 min at 37°C, cells were fixed 3 times with methanol and acetic acid (3:1) and fixed cells were spread on slides.

Multicolor fluorescence in situ hybridization (mFISH) was performed using commercially available mFISH kits (MetaSystems, Altlussheim, Germany) according to the manufacturer’s protocol. Briefly metaphase spreads were hardened 70°C for 2 h. After applying mFISH probes on the metaphase spreads, co-denaturation of target DNA with probe DNA was performed at 70°C for 5 min, followed by 72 h incubation at 37°C to allow hybridization of the probes. The slides were then washed twice with 50% formamide/2 × standard saline citrate (SSC) solution for 20 min at 37°C, 2 × SSC for 10 min at room temperature and 1 × SSC for 10 min. The slides were then counterstained with 4′,6-diamidino-2-phenylindole (DAPI) and mounted. Separate fluorochrome images were captured using a Leica DC 350FX cooled CCD camera (Leica) mounted on a Leica DMRA2 microscope using Leica CW4000 FISH software. The images were analyzed using Leica CW4000 karyo (Leica).

## Results

### Cell morphology and colony formation in 2D and 3D culture models

**Figure 3:**
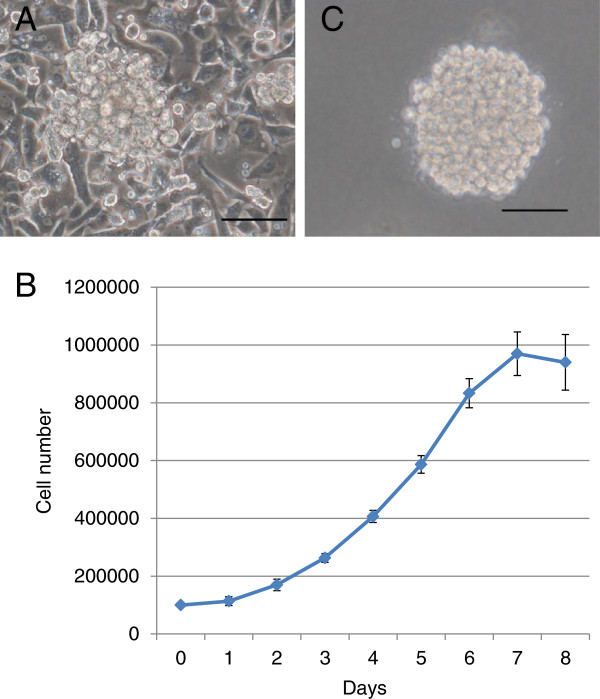
**Characteristics of Asra-EPS cells *in vitro*. (A)** Morphology of Asra-EPS cells under 2D culture by phase-contrast microscopy. **(B)** Growth curve of Asra-EPS cells *in vitro*. Each point represents the mean value ± standard deviation (SD) (n=3). **(C)** Colony formation of Asra-EPS cells in soft agar. Scale bars: 100μm.

The cell line Asra-EPS has now been growing in standard 2D culture without interruption for over 15 months and has been passed at least 150 times. The line appeared to contain epithelial-like polygonal cells but, in addition, more mesenchymal-like spindle-shaped cells were apparent (Figure 3A). Both of these morphological phenotypes appeared to be stable with repeated passaging. Asra-EPS cells showed excellent growth without contact inhibition. The doubling time of Asra-EPS cells in logarithmic growth phase was approximately 38 h (Figure 3B). Asra-EPS cells could form colonies in soft agar (Figure 3C).

### Tumorigenicity of Asra-EPS cells in nude mice

**Figure 4:**
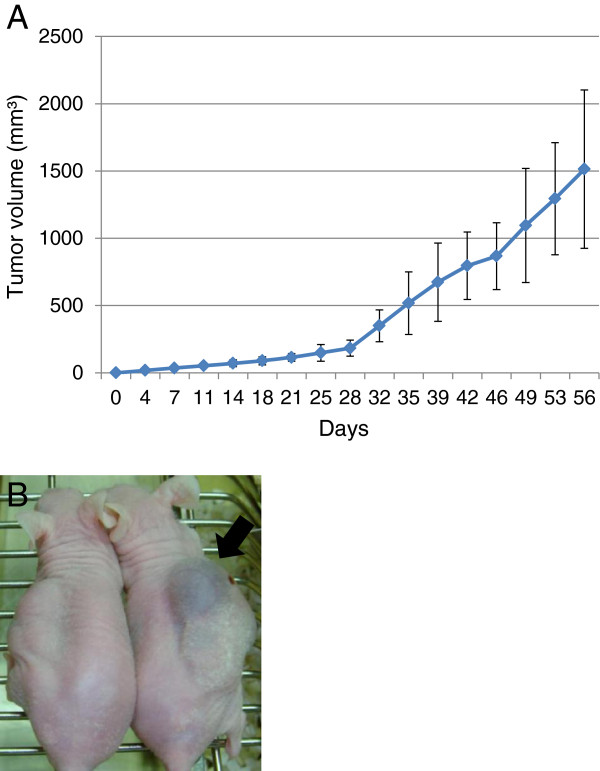
**Characteristics of Asra-EPS tumors *in vivo*. (A)** Growth curve of Asra-EPS tumors in nude mice. Each point represents the mean value ± SD (n=5). **(B)** Asra-EPS tumors with large cysts containing hemorrhagic fluid (black arrow).

Fourteen days after injection of 1 × 10^7^ Asra-EPS cells into the nude mice, tumor growth was evident in all 5 mice tested. The efficiency of tumor formation was extremely high. These tumors were serially transplantable in further mice as a stable xenograft line. The tumors were then resected, digested, and plated into dishes. These re-plated cells showed similar morphological features to the original cells from the patient tumor tissues. Eight weeks after injection of Asra-EPS cells, the average of Asra-EPS tumor volume was 1514 mm^3^ (Figure 4A). Asra-EPS tumors formed in mice also exhibited blood-filled cyst formation within them as they grew largely, reflecting the clinical feature of the original tumor (Figure 4B).

### Morphologic characteristics of Asra-EPS tumors

**Figure 5:**
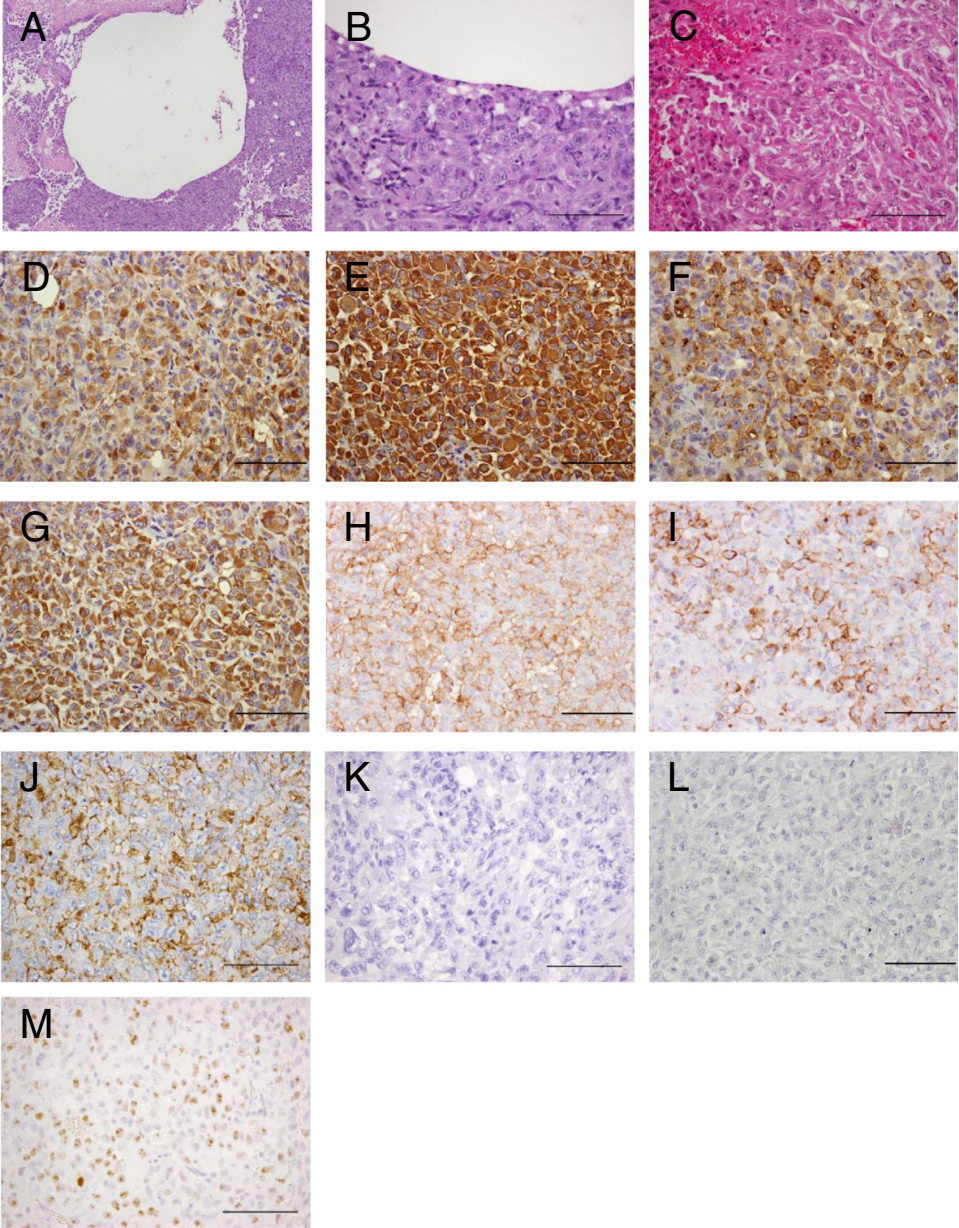
**Microscopic features of Asra-EPS tumors formed in nude mice. (A-C)** Histological appearance of Asra-EPS tumors **(H&E)**. Histological features of Asra-EPS tumors were almost the same as those of the original tumor. **(D-M)** Immunohistochemical reactivity in Asra-EPS tumors. Tumor cells were diffusely positive for AE1/AE3 **(D)**, CAM5.2 **(E)**, EMA **(F)**, vimentin **(G)**, CD31 **(H)**, CD34 **(I)** and CA 125 **(J)**, but negative for INI-1 **(K)** and factor VIII-related antigen **(L)**. Positive rate of Ki-67 was approximately 50% (M). Scale bars: 100μm.

**Figure 6:**
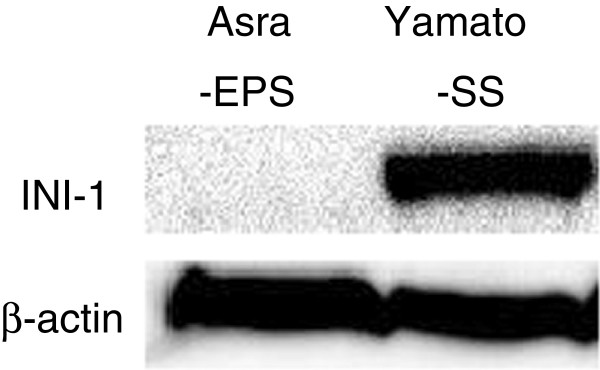
No INI-1 expression of Asra-EPS.

H&E staining of Asra-EPS xenograft tumors with cysts formation and a wide range of hemorrhage demonstrated histological appearances resembling the original tumor (Figure 5A, B, C). The immunohistochemical profiles of both tissues from the patient tumor tissues and from Asra-EPS tumors formed in mice were the same as previous studies on human angiomatoid ES tissues [8,9]. Asra-EPS tumors exhibited positive staining for AE1/AE3 (Figure 5D), CAM5.2 (Figure 5E), EMA (Figure 5F), vimentin (Figure 5G), CD31 (Figure 5H), CD34 (Figure 5I) and CA 125 (Figure 5J), but negative staining for INI-1 (Figure 5K) and factor VIII-related antigen (Figure 5L). Positive rate of Ki-67 in xenografted tumor was similar to that in the patient’s tumor, approximately 50% (Figure 5M). In western blotting, no INI-1 expression was found in Asra-EPS cells, in contrast to the synovial sarcoma cell line Yamato-SS [22], which was used as a control (Figure 6).

### Secretion of CA 125, IL-6 and VEGF by Asra-EPS cells *in vitro*

Amounts of CA 125, IL-6 and VEGF secreted into the culture medium of 1 × 10^5^ Asra-EPS cells at 7th day culture were as follows: CA 125 145 U/ml; IL-6 7760 pg/ml; VEGF 957 pg/ml. Asra-EPS cells produced CA 125, IL-6 and VEGF at high levels.

### Chromosome analysis

**Figure 7:**
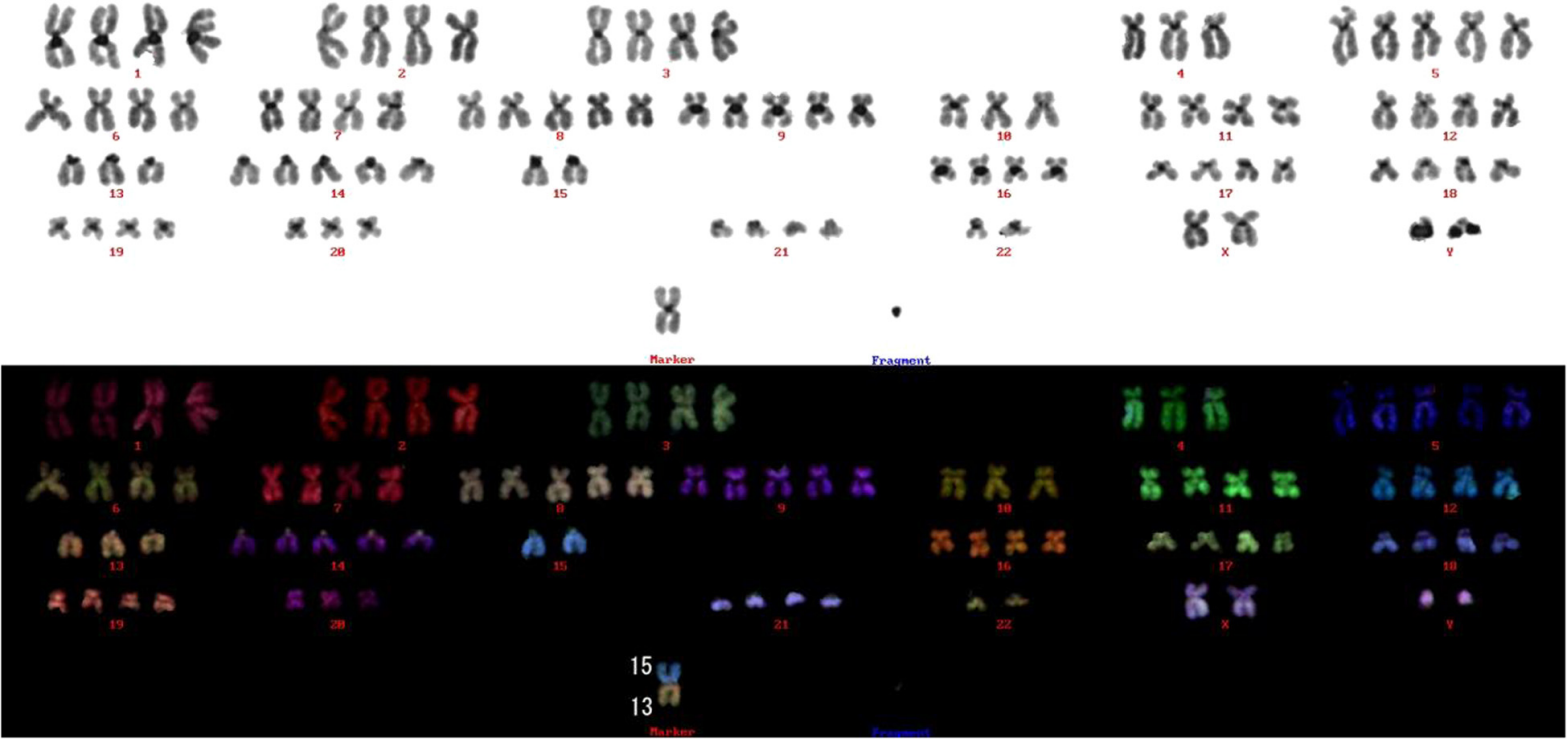
**Representative karyotype of Asra-EPS at the 30th passage.** It is 90, XXYY, -4, +5, +8, +9, -10, -13, t(13;15), +14, -15, -15, -20, -22, -22, +1mar.

In the chromosomal analysis, 50 metaphases were examined. The chromosome number of Asra-EPS cells at the 30th passage ranged from 45 to 133, with a mode of 90. The karyotype of Asra-EPS showed near-tetraploidy with some chromosomal translocations and fragments (Figure 7). No recurrent chromosomal translocation was detected.

## Discussion

Angiomatoid ES is less common variant of ES and its clinical features are still unknown [8,9]. Histologically, the tumor of angiomatoid ES is composed of epithelioid and spindle-shaped tumor cells exhibiting intracytoplasmic vacuoles reminiscent of intracytoplasmic vascular lumen formation [23]. The morphology of the tumor cells is similar to that seen in classical ES. Angiomatoid ES shows prominent areas of hemorrhage and foci with blood-filled cystic spaces within the tumor [8,23]. These features closely resemble those seen in malignant vascular tumors with epithelioid cytomorphology, including epithelioid angiosarcoma and epithelioid hemangioendothelioma [24].

In this study, we have succeeded in establishing an angiomatoid ES cell line, called Asra-EPS. To the best of our knowledge, this is the first report of establishing and characterizing an angiomatoid ES cell line. Asra-EPS cells exhibited the morphology of epithelial-like polygonal cells and mesenchymal-like spindle-shaped cells. Asra-EPS cell line had epithelial and mesenchymal features similar to those of the original tumor as revealed by immunohistochemistry, secreted CA 125, IL-6 and VEGF, did not express INI-1, and exhibited tumorigenicity in nude mice.

CA 125 is a tumor antigen forming the basis for a serum assay widely used to monitor the clinical status of patients with ovarian cancer [25,26]. Recently, elevated serum CA 125 was reported in two patients with ES [27]. Furthermore, a subsequent study by the same group indicated that 10 of their 11 cases of ES (classic type: 9 cases; proximal type: 1 case) showed strong immunoreactivity for CA 125 in the tumor cells [28]. Therefore, CA 125 could be considered to be a generally useful tumor marker for diagnosing ES and monitoring its clinical course. Nevertheless, it is unknown whether CA 125 would be a useful marker for diagnosing and monitoring the course of angiomatoid ES.

IL-6 is presently considered as the most important mediator of acute-phase protein synthesis in hepatocytes and cell culture experiments have suggested that IL-6 stimulates the synthesis of serum CRP [29-31]. A classical ES cell line, designated as ES-OMC-MN, was reported to produce IL-6, but not to be tumorigenic in nude mice [17]. In this study, we showed that Asra-EPS could constitutively produce CA 125 and IL-6. In this case, blood analyses showed significantly high levels of CA 125 and CRP in the sera before the surgery and rapid decline of those levels to the normal range after the surgery. At the recurrence, the serum levels of CA 125 and CRP were re-elevated. Taken together, these clinical findings indicated that the measurement of serum CA 125 and CRP could be recommended to diagnose angiomatoid ES and monitor its clinical course.

INI-1 is the protein product of the *hSNF5/INI1/SMARCB1/BAF47* gene as a tumor suppressor gene located on 22q11.2 that is characteristically inactivated in malignant rhabdoid tumor (MRT) of infancy [32-35]. In the previous study, most ES cases, both classical and proximal type, were reported to show complete loss of expression of INI-1 [36]. The present study demonstrated that INI-1 protein expression was also lost in the original tumor as well as our established angiomatoid ES cell line. INI-1 expression is retained in most of epithelioid angiosarcoma and epithelioid hemangioendothelioma. Thereby, the immunohistochemical profile of the angiomatoid ES was useful to differentiate them from malignant epithelioid vascular tumors with INI-1 and CA 125 immunoreactivity [21]. Asra-EPS cell line might provide us with a research material to elucidate the mechanism in the loss of INI-1 protein expression and its oncogenic functions in ES pathogenesis.

Surprisingly, Asra-EPS tumors formed in nude mice also showed prominent areas of hemorrhage and foci with blood-filled cystic spaces as they grew largely. Their pathological appearance of cystogenesis in Asra-EPS closely resembles that of the original tumor. While OVCAR-3, one of ovarian cancer cell lines, has no potential to form the cysts itself [37], Duyndam and colleague reported that VEGF_165_-overexpression induced OVCAR-3 xenograft tumors to form the cysts containing hemorrhagic fluid and that VEGF_165_ might play a role in the cystogenesis [38]. To our knowledge, there is no report of cancer or sarcoma cell lines to spontaneously generate cysts within the xenograft tumors *in vivo,* except for the above described genetically engineered cancer cell line. Therefore, Asra-EPS is the first sarcoma cell line, with the potential to form tumors with large cysts containing hemorrhagic fluid in nude mice. Since Asra-EPS secreted a considerable amount of VEGF, molecular target drugs against VEGF might be effective to suppress the cystogenesis in Asra-EPS xenograft tumors. However, bevacizumab (Genentech/Roche, South San Francisco, CA, USA), a humanized monoclonal antibody to human VEGF, could not inhibit the formation of cysts in Asra-EPS tumors (data not shown). Collectively, these findings suggested that other growth factors than VEGF or cytokines might contribute to the cystogenesis in Asra-EPS tumors and further investigation is needed to clarify the molecular mechanism of cyst formation.

## Conclusions

A newly established Asra-EPS cell line fully preserved the morphological and biochemical characteristics of the original tumor diagnosed as angiomatoid ES. Asra-EPS is an excellent tool for further investigation of the nature of angiomatoid ES, the mechanism in the formation of cyst, and potential therapies for patients with ES.

## Abbreviations

BSA: Bvine serum albumin; CA 125: Cancer antigen 125; CLEIA: Chemiluminescent enzyme immunoassay; CRP: C-reactive protein; CT: Computed tomography; DAB: 3,3′-diaminobenzidine tetrahydrochlorid; DAPI: 4′6-diamidino-2-phemylindole; DMEM: Dulbecco’s modified Eagle’s medium; ECL: Enhanced chemiluminescence; EMA: Epithelial membrane antigen; ES: Epithelioid sarcoma; FBS: Fetal bovine serum; H&E: Hematoxylin and eosin; HRP: Horseradish peroxidase; IL-6: Interleukin-6; INI-1: Integrase interactor 1; mFISH: multicolor fluorescence in situ hybridization; MRI: Magnetic resonance imaging; MRT: Malignant rhabdoid tumor; PBS: Phosphate-buffered saline; SSC: Standard saline citrate; TBS-T: Tris-buffered saline containing 0.1% Tween; VEGF: Vascular endothelial growth factor.

## Competing interests

The authors declare that they have no competing interests.

## Authors’ contributions

YI carried out the whole study and drafted the manuscript. YI and NN designed the study. NN revised the manuscript. NN, ST and RO operated the patient. NN, RO and MK treated the patient. HO, HY, ST, KH, AM and HY helped to draft the manuscript. KY and EM performed the pathological examination. All authors read and approved the final manuscript.

## References

[B1] Enzinger FM Epithelioid sarcoma. A sarcoma simulating a granuloma or carcinoma Cancer 1970 26 1029 1041 10.1002/1097-0142(197011)26:5<1029::AID-CNCR2820260510>3.0.CO;2-R 5476785

[B2] de Visscher SA van Ginkel RJ Wobbes T Veth RP Ten Heuvel SE Suurmeijer AJ Hoekstra HJ Epithelioid sarcoma: still an only surgically curable disease Cancer 2006 107 606 612 10.1002/cncr.22037 16804932

[B3] Chase DR Enzinger FM Epithelioid sarcoma: diagnosis, prognostic indicators, and treatment Am J Surg Pathol 1985 9 241 263 10.1097/00000478-198504000-00001 4014539

[B4] Halling AC Wollen PC Pritchard DJ Vlasak R Nascimento AG Epithelioid sarcoma: a clinicopathologic review of 55 cases Mayo Clin Med 2004 71 636 642 10.1016/S0025-6196(11)63000-0 8656704

[B5] Guillou L Wadden C Coindre JM Krausz T Fletcher CD “Proximal-type” epithelioid sarcoma: a distinctive aggressive neoplasm showing rhabdoid features, clinicopathologic, immunohistochemical, and ultrastructural study of a series Am J Surg Pathol 1997 21 130 146 10.1097/00000478-199702000-00002 9042279

[B6] Von Hochstetter AR Meyer VE Grant JW Honegger HP Schreiber A Epithelioid sarcoma mimicking angiosarcoma: the value of immunohistochemistry in the differential diagnosis Virchows Arch A Pathol Anat Histopathol 1991 418 271 278 10.1007/BF01606067 1900974

[B7] Mirra JM Kessler S Bhuta S Eckardt J The fibroma-like variant of epithelioid sarcoma. A fibrohistiocytic/myoid cell lesion often confused with benign and malignant spindle cell tumors Cancer 1992 69 1382 1395 10.1002/1097-0142(19920315)69:6<1382::AID-CNCR2820690614>3.0.CO;2-Y 1371711

[B8] Kaddu S Wolf I Horn M Kerl H Epithelioid sarcoma with angiomatoid features: report of an unusual case arising in an elderly patient within a burn scar J Cutan Pathol 2008 35 324 328 10.1111/j.1600-0560.2007.00802.x 18251749

[B9] Brassesco MS Valera ET Castro-Gamero AM Moreno DA Silveira TP Mori BM Engel EE Scrideli CA Tone LG Cytogenetic findings in an epithelioid sarcoma with angiomatoid features. A case report Genet Mol Res 2009 8 1211 1217 10.4238/vol8-4gmr656 19866439

[B10] Reeves BR Fisher C Smith S Courtenay VD Robertson D Ultrastructural, immunocytochemical and cytogenetic characterization of a human epithelioid sarcoma cell line (RM-HS1) J Natl Cancer Inst 1987 78 7 18 2432306 10.1093/jnci/78.1.7

[B11] Kelland LR Bingle L Radiosensitivity and characterization of a newly established cell line from an epithelioid sarcoma Br J Cancer 1988 58 322 325 10.1038/bjc.1988.211 3179184 PMC2246596

[B12] Gerharz CD Moll R Ramp U Mellin W Gabbert HE Multidirectional differentiation in a newly established human epithelioid sarcoma cell line (GRU-1) with co-expression of vimentin, cytokeratin and neurofilament proteins Int J Cancer 1990 45 143 152 10.1002/ijc.2910450126 1688830

[B13] Roche H Julia AM Jozan S Muller C Dastugue N Arquier MA Marques B Laurent G Canal P Characterization and chemosensitivity of a human epithelioid sarcoma cell line (SARCCR 2) Int J Cancer 1993 54 663 668 10.1002/ijc.2910540423 8099901

[B14] Sonobe H Furihata M Iwata J Oka T Ohtsuki Y Hamasato S Fujimoto S Morphological characterization of a new human epithelioid sarcoma cell line, ES020488, *in vitro* and *in vivo* Virchows Arch B Cell Pathol 1993 63 219 225 10.1007/BF02899265 7685133

[B15] Helson C Melamed M Braverman S Traganos F Preti R Helson L VA-ES-BJ: an epithelioid sarcoma cell line Int J Oncol 1995 7 51 56 21552805 10.3892/ijo.7.1.51

[B16] Iwasaki H Ohjimi Y Ishiguro M Isayama T Kaneko Y Yoh S Emoto G Kikuchi M Epithelioid sarcoma with an 18q aberration Cancer Genet Cytogenet 1996 91 46 52 10.1016/S0165-4608(95)00315-0 8908166

[B17] Kusakabe H Sakatani S Yonebayashi K Kiyotake K Establishment and characterization of an epithelioid sarcoma cell line with an autocrine response to interleukin-6 Arch Dermatol Res 1997 289 224 233 10.1007/s004030050184 9143739

[B18] Goto H Takahashi H Funabiki T Ikuta K Sasaki H Nagashima Y Neural differentiation of a novel cell line, YCUS-5, established from proximal-type epithelioid sarcoma of a child Med Pediatr Oncol 1999 33 137 138 10.1002/(SICI)1096-911X(199908)33:2<137::AID-MPO18>3.0.CO;2-N 10398195

[B19] Nishio J Iwasaki H Nabeshima K Ishiguro M Naumann S Isayama T Naito M Kaneko Y Kikuchi M Bridge JA Establishment of a new human epithelioid sarcoma cell line, FU-EPS-1: molecular cytogenetic characterization by use of spectral karyotyping and comparative genomic hybridization Int J Oncol 2005 27 361 369 16010416

[B20] Hoshino M Kawashima H Ogose A Kudo N Ariizumi T Hotta T Umezu H Hatano H Morita T Nishio J Iwasaki H Endo N Serum CA 125 expression as a tumor marker for diagnosis and monitoring the clinical course of epithelioid sarcoma J Cancer Res Clin Oncol 2010 136 457 464 10.1007/s00432-009-0678-1 19756736 PMC11827905

[B21] Sakharpe A Lahat G Gulamhusein T Liu P Bolshakov S Nguyen T Zhang P Belousov R Young E Xie X Rao P Hornick JL Lazar AJ Pollock RE Lev D Epithelioid sarcoma and unclassified sarcoma with epithelioid features: clinicopathological variables, molecular markers, and a new experimental model Oncologist 2011 16 512 522 10.1634/theoncologist.2010-0174 21357725 PMC3228127

[B22] Naka N Takenaka S Araki N Miwa N Hashimoto N Yoshioka K Joyama S Hamada K Tsukamoto Y Tomita Y Ueda T Yoshikawa H Itoh K Synovial sarcoma is stem cell malignancy Stem Cells 2010 28 1119 1131 20518020 10.1002/stem.452

[B23] Miettinen M Fanburg-Smith JC Virolainen M Shmookler BM Fetsch JF Epithelioid sarcoma. An immunohistochemical analysis of 112 classical and variant cases and a discussion of the differential diagnosis Hum Pathol 1999 30 934 942 10.1016/S0046-8177(99)90247-2 10452506

[B24] Von Hochstetter AR Meyer VE Grant JW Honegger HP Schreiber A Epithelioid sarcoma mimicking angiosarcoma. The value of immunohistochemistry in the differential diagnosis Virchows Arch Pathol Anat Histopathol 1991 418 271 278 10.1007/BF01606067 1900974

[B25] Yin BW Dniatrian A Lloyd KO Ovarian cancer antigen CA125 is encoded by the MUC16 mucin gene Int J Cancer 2002 98 737 740 10.1002/ijc.10250 11920644

[B26] Bast RC Jr Badgwell D Lu Z Marquez R Rosen D Liu J Baggerly KA Atkinson EN Skates S Zhang Z Lokshin A Menon U Jacobs I Lu K New tumor markers: CA125 and beyond Int J Gynecol Cancer 2005 15 274 281 10.1111/j.1525-1438.2005.00441.x 16343244

[B27] Kato H Hatori M Watanabe M Kokubun S Epithelioid sarcoma with elevated serum CA125: report of two cases Jpn J Clin Oncol 2003 33 141 144 10.1093/jjco/hyg030 12672792

[B28] Kato H Hatori M Kokubun S Watanabe M Smith RA Hotta T Ogose A Morita T Murakami T Aiba S CA125 expression in epithelioid sarcoma Jpn J Clin Oncol 2004 34 149 154 10.1093/jjco/hyh027 15078911

[B29] Shine B de Beer FC Pepys MB Solid phase radioimmunoassays for human C-reactive protein Clin Chim Acta 1981 117 13 23 10.1016/0009-8981(81)90005-X 7333010

[B30] de Beer FC Hind CR Fox KM Allan RM Maseri A Pepys MB Measurement of serum C-reactive protein concentration in myocardial ischaemia and infarction Br Hearl J 1982 47 239 243 10.1136/hrt.47.3.239 PMC481128 7059401

[B31] Castell JV Gomez-Lechon ML David M Andus T Geiger T Trullenque R Fabra R Heinrich PC Interleukin-6 is the major regulator of acute phase protein synthesis in adult human hepatocytes FEBS Lett 1989 242 237 239 10.1016/0014-5793(89)80476-4 2464504

[B32] Versteege I Sevenet N Lange J Rousseau-Merck MF Ambros P Handgretinger R Aurias A Delattre O Truncating mutations of hSNF5/INI1 in aggressive paediatric cancer Nature 1998 394 203 206 10.1038/28212 9671307

[B33] Bourdeaut F Freneaux P Thuille B Lellouch-Tubiana A Nicolas A Pierron G Sainte-Rose C Bergeron C Bouvier R Rialland X Laurence V Michon J Sastre-Garau X Delattre O hSNF5/INI1-deficient tumours and rhabdoid tumours are convergent but not fully overlapping entities J Pathol 2007 211 323 330 10.1002/path.2103 17152049

[B34] Kalpana GV Marmon S Wang W Crabtree GR Goff SP Binding and stimulation of HIV-1 integrase by a human homolog of yeast transcription factor SNF5 Science 1994 266 2002 2006 10.1126/science.7801128 7801128

[B35] Ae K Kobayashi N Sakuma R Ogata T Kuroda H Kawaguchi N Shinomiya K Kitamura Y Chromatin remodeling factor encoded by ini1 G1 arrest and apotosis in ini1-deficient cells Oncogene 2002 21 3112 3120 10.1038/sj.onc.1205414 12082626

[B36] Hornick JL Dal Cin P Fletcher CD Loss of INI1 expression is characteristic of both conventional and proximal-type epithelioid sarcoma Am J Surg Pathol 2009 33 542 550 10.1097/PAS.0b013e3181882c54 19033866

[B37] Hamilton TC Young RC McKoy WM Grotzinger KR Green JA Chu EW Whang-Peng J Rogan AM Green WR Ozols RF Characterization of a human ovarian carcinoma cell line (NIH:OVCAR-3) with androgen and estrogen receptors Cancer Res 1983 43 5379 5389 6604576

[B38] Duyndam MC Hilhorst MC Schluper HM Verheul HM van Diest PJ Kraal G Pinedo HM Boven E Vascular endotherial growth factor-165 overexpression stimulates angiogenesis and induces cyst formation and macrophage infiltration in human ovarian cancer xenografts Am J Pathol 2002 160 537 548 10.1016/S0002-9440(10)64873-0 11839574 PMC1850657

